# Redescription of *Bakuella (Bakuella) marina* Agamaliev and Alekperov, 1976 (Protozoa, Hypotrichia), With Notes on Its Morphology, Morphogenesis, and Molecular Phylogeny

**DOI:** 10.3389/fmicb.2021.774226

**Published:** 2022-02-10

**Authors:** Rong Zhu, Qi Zhang, Lan Tang, Yan Zhao, Jingbao Li, Fengchao Li

**Affiliations:** ^1^College of Life Sciences, Hebei University, Baoding, China; ^2^College of Life Sciences, Capital Normal University, Beijing, China; ^3^Key Laboratory for Space Bioscience and Biotechnology, School of Life Sciences, Institute of Special Environmental Biophysics, Northwestern Polytechnical University, Xi’an, China; ^4^Innovation Center for Bioengineering and Biotechnology of Hebei Province, Baoding, China

**Keywords:** phylogenetic analysis, Hypotrichia, *Bakuella (Bakuella) marina*, SSU rDNA, ontogenesis

## Abstract

Because the original description of *Bakuella (Bakuella) marina*, type of the genus, is only based on protargol-impregnated specimens, one of the important living features, namely, the presence/absence of cortical granules, remains unknown so far. In the present work, a detailed investigation of a Chinese population of *B. (Bakuella) marina* is carried out using the integrated approaches, and the live morphology, ontogenesis, and molecular information of *B. (Bakuella) marina* are presented for the first time. The infraciliature of this population corresponds perfectly with that of the original description. The *in vivo* observation indicates that *B. (Bakuella) marina* possesses colorless cortical granules. The most prominent morphogenetic feature of *B. (Bakuella) marina* is that the parental adoral zone of membranelles is completely replaced by the newly formed one of the proters. Molecular phylogenetic analysis based on a small subunit ribosomal gene (SSU rDNA) shows that five *Bakuella* species are clustered with the species from other six Urostylid genera, namely, *Anteholosticha*, *Apobakuella*, *Diaxonella*, *Holosticha*, *Neobakuella*, and *Urostyla*. The monophyletic probabilities of the family Bakuellidae, genus *Bakuella*, subgenus *B. (Bakuella)*, and subgenus *B. (Pseudobakuella)* are rejected by the approximately unbiased test. This study further shows that the family Bakuellidae, genus *Bakuella*, and subgenus *B. (Bakuella)* are all nonmonophyletic groups. In order to establish a reasonable classification system, information on molecular and morphogenesis of more Bakuellids and its related species is urgently needed.

## Introduction

The ciliated protozoa are a large and diverse group, which live in a wide variety of habitats (Kaur et al., 2019; Kim and Min, 2019; Luo et al., 2019; Shao et al., 2019, 2020; Bai et al., 2020; Wu et al., 2020; Liu et al., 2021; Wang et al., 2021). Hypotrichs are the most complex, highly differentiated and speciose group of ciliates, and more than 1,000 valid species have been found in this group (Foissner, 2016; Berger, 2018; Foissner and Berger, 2021). Recent researches show that in addition to morphological characteristics, ontogeny and molecular information is also important for the construction of the systematic relationship among hypotrich ciliates (Chen et al., 2019; Kim et al., 2019; Moon et al., 2019; Wang et al., 2019, 2020; Zhang et al., 2019, 2020; Zhu et al., 2019; Lian et al., 2020; Park et al., 2020; Xu et al., 2020). Regretfully, although a lot of research work has been done on the ontogeny and molecular phylogeny of hypotrichs in the past 30 years, because of various reasons, there are still a large number of species that lack of ontogeny and molecular information (Berger, 1999, 2006, 2008, 2011).

*Bakuella* was established by Agamaliev and Alekperov (1976). It is the type genus of the speciose family Bakuellidae (Jankowski, 1979). Berger (2006) revised the genus *Bakuella* and gave the following diagnosis: adoral zone of membranelles (AZM) continuous, three more or less distinctly enlarged frontal cirri, more than one buccal cirrus, two or more frontoterminal cirri, midventral complex comprising midventral pairs and midventral rows, transverse cirri present, one left and one right marginal cirral row, caudal cirri absent. Further, Berger (2006) divided *Bakuella* into two subgenera, *Bakuella (Bakuella)* and *B. (Pseudobakuella)*, based on the number of frontoterminal cirri (the former has more than two frontoterminal cirri vs. only two in the later). Currently, there are 15 species assigned in this genus (Song et al., 1992; Berger, 2006; Kumar et al., 2010; Chen et al., 2013; Jo et al., 2015; Li et al., 2021; Ma et al., 2021). Among them, molecular data (SSU rDNA) are reported for six members, namely, *B. (Bakuella) granulifera*, *B. (Bakuella) incheonensis*, *B. (Bakuella) subtropica*, *B. (Bakuella) xianensis*, *B. (Pseudobakuella) guangdongica*, and *B. (Pseudobakuella) litoralis*, and six species, including *B. (Bakuella) edaphoni*, *B. (Bakuella) granulifera*, *B. (Bakuella) pampinaria*, *B. (Bakuella) subtropica*, *B. (Pseudobakuella) guangdongica*, and *B. (Pseudobakuella) salinarum*, are available for detailed morphogenetic information (Eigner and Foissner, 1992; Song et al., 1992; Berger, 2006; Chen et al., 2013, 2020b; Li et al., 2021). However, the morphogenetic characteristics, *in vivo* morphology and molecular information of *Bakuella (Bakuella) marina*, and type species of the genus have not been described so far.

**FIGURE 1:**
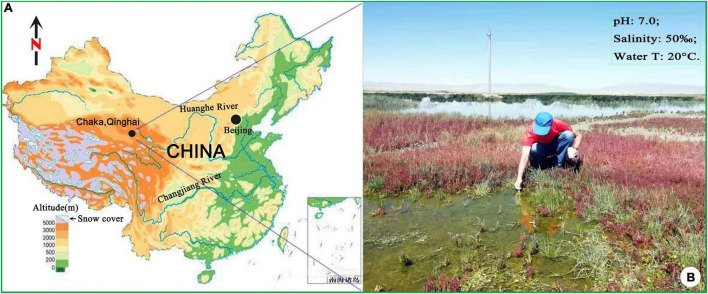
Sampling location and surrounding areas **(A,B)**.

In the summer of 2019, a urostylid ciliate was collected from a hypersaline wetland near the bank of Chaka Salt Lake on Qinghai-Tibet Plateau, China (Figures 1A,B). After a detailed morphological comparison with previous populations, we confirmed this isolation to be *B. (Bakuella) marina*. The diagnosis of this species was improved based on Chinese population and the previous data. In view of the importance of morphogenetic and molecular information on both the species identification and the phylogenetic analysis of hypotrichs (Bharti et al., 2019; Jung and Berger, 2019; Chen et al., 2020a), detailed morphogenetic process and molecular data (SSU rDNA) of *B. (Bakuella) marina* were also provided in the present article.

## Materials and Methods

### Sampling, Cultivation, and Isolation

The water sample (salinity 50‰, pH 7.0) was collected in August 2019, from a wetland near the bank of Chaka Salt Lake on Qinghai-Tibet Plateau, China (36°45′36″ N, 99°04′50″ E). The temperature of the sampling water is 20°C, and the altitude of the sampling site is 3,059 m. In the laboratory, the clonal culture of *B. (Bakuella) marina* was established at room temperature (approximately 25°C) in sterilized salt water (salinity 50‰). Two or three autoclaved rice grains were added to enrich the bacteria as food.

### Morphological and Ontogenetic Investigations

Live observations were carried out by using a bright-field microscope equipped with differential interference contrast. The protargol silver staining method of Wilbert (1975) was used to reveal the infraciliature and the nuclear apparatus. The protargol used in this study was synthesized with reference to the method of Pan et al. (2013). Drawings of stained specimens were conducted at a magnification of 1,000× with the aid of a camera lucida. Measurements were made with an ocular micrometer. To illustrate the changes that occur during ontogenetic processes, ciliary structures of parental cells were depicted by contours, whereas those of the daughter cells were shaded black. The terminology used herein is informed by that of Berger (2006) and Song and Shao (2017).

### DNA Extraction, Polymerase Chain Reaction Amplification, and Gene Sequencing

Total genomic DNA of *B. (Bakuella) marina* was extracted from a single cell using the DNeasy Blood & Tissue Kit (Qiagen GmbH, Germany), according to the manufacturer’s instruction and previous studies (Li et al., 2019). Polymerase chain reaction (PCR) amplifications of SSU rDNA were performed with universal eukaryotic primers 18S-F (5′-AAC CTG GTT GAT CCT GCC AGT-3′) and 18S-R (5′-TGA TCC TTC TGC AGG TTC ACC TAC-3′) (Medlin et al., 1988). The amplification conditions for SSU rDNA were as follows: a prerun of 30 s at 98°C, followed by 35 cycles consisting of denaturation at 98°C for 30 s, annealing at 60°C for 20 s, and extension at 72°C for 1 min. After 35 cycles, the final extension step was run at 72°C for 2 min. The PCR products were sequenced in both directions, which was carried out by BGI gene sequencing company in Shenzhen. And three internal primers, 900S-F (5′-CGA TCA GAT ACC GTC CTA GT-3′), 900S-R (5′-ACT AGG ACG GTA TCT GAT CG-3′), and B (5′-AAY CTG GTT GAT YYT GCC AG-3′), were used as sequencing primers.

### Phylogenetic Analyses

**FIGURE 2:**
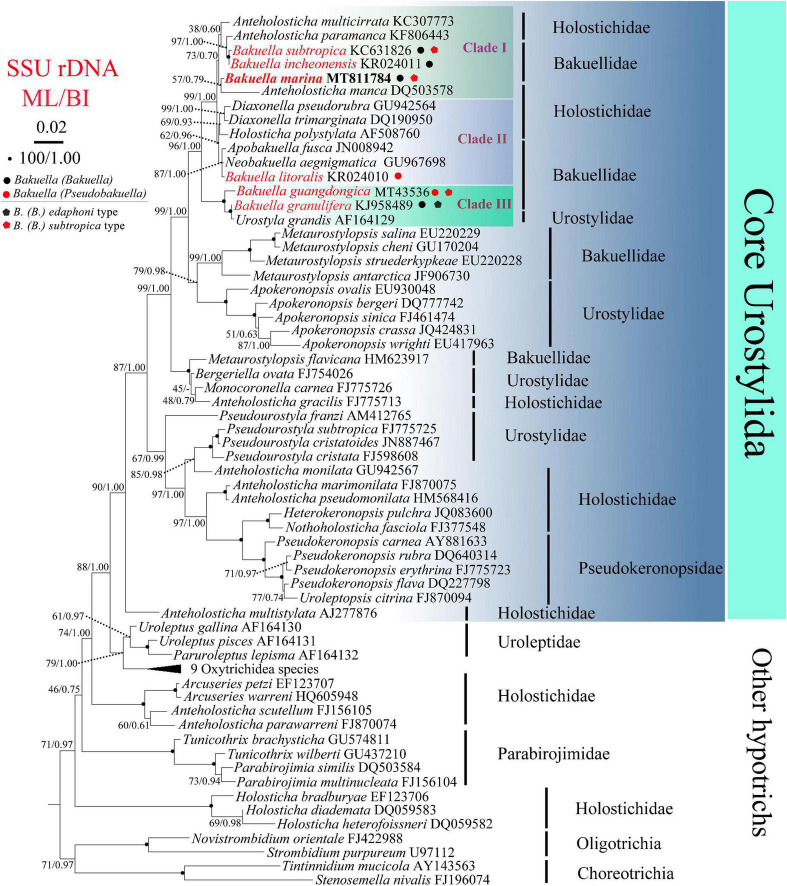
Maximum likelihood (ML) and Bayesian inference (BI) phylogenetic trees derived from SSU rDNA sequences. Species from Choreotrichia and Oligotrichia were chosen as outgroup. Numbers at the nodes show the bootstrap values for ML and BI (ML/BI). “–” refers to disagreement in topologies of the ML and BI trees, and thus only the bootstrap values of ML are presented. The scale bar corresponds to two substitutions per 100 nucleotide positions (0.02).

Except the newly sequenced SSU rDNA of *B. (Bakuella) marina*, all of the sequences used in the phylogenetic analyses were obtained from the GenBank database (for accession numbers, see Figure 2). *Novistrombidium orientale*, *Stenosemella nivalis*, *Strombidium purpureum*, and *Tintinnidium mucicola* were used as outgroups. Sequences were aligned using Clustal W implemented in BioEdit 7.0.9.1 with default parameters (Hall, 1999). Regions that could not be aligned unambiguously were removed, and ends were trimmed manually, resulting in a final matrix of 1,577 characters. The program MrModeltest 2.2 (Nylander, 2004) selected the GTR + *I* (= 0.6435) + *G* (= 0.8618) as the best model with the Akaike Information Criterion, which was then used for maximum likelihood (ML) and Bayesian inference (BI) analyses on the CIPRES Science Gateway (Miller et al., 2010). ML analysis was performed with RAxML-HPC2 (8.2.12) on XSEDE (Stamatakis et al., 2008). BI analysis was carried out using MrBayes (3.2.7a) on XSEDE (Ronquist and Huelsenbeck, 2003). The Markov chain Monte Carlo was run for 1,000,000 generations and sampled once every 1,000 generations, with the first 25% of trees as burn-in. Seaview v.4.3.3 (Gouy et al., 2010) and MEGA 4.0 (Tamura et al., 2007) were used to visualize tree topologies. The approximately unbiased (AU) test was implemented using CONSEL v 0.1 (Shimodaira and Hasegawa, 2001) to test the probabilities of the monophyly of the family Bakuellidae, the genus *Bakuella*, and the subgenus *B. (Bakuella)*.

## Results

### Small Subunit Ribosomal Gene Sequence and Phylogenetic Analyses

The SSU rDNA sequence of *B. (Bakuella) marina* has been deposited in the GenBank database under the accession number MT811784. The length and GC content are 1,577 bp and 44.71%, respectively. The topologies of the ML and BI trees are similar, and therefore only the ML tree is shown. According to the phylogenetic analyses of the 70-taxon alignment, *B. (Bakuella) marina* and other five congeners were distributed in three separate clades that were nested within the core Urostylida (Figure 2). In clade I, *B. (Bakuella) marina* first grouped with *Anteholosticha manca* with moderate support (57% ML, 0.79 BI) and then clustered with a subclade containing two *Bakuella* species (*B. (Bakuella) incheonensis* and *B. (Bakuella) subtropica*) and two *Anteholosticha* species (*A. paramanca* and *A. multicirrata*) with moderate support (73% ML, 0.70 BI). In clade II, *B. (Pseudobakuella) litoralis* formed a polytomy with *Neobakuella aenigmatica* and *Apobakuella fusca* with high support (87% ML, 1.00 BI) and then clustered with a subclade containing *Holosticha polystylata* and two *Diaxonella* species (*D. pseudorubra* and *D. trimarginata*) with moderate support (62% ML, 0.96 BI). In clade III, *B. (Bakuella) granulifera* first clustered with *Urostyla grandis* with full support and then clustered with *B. (Pseudobakuella) guangdongica* with full support. The probabilities of the monophyly of the family Bakuellidae, the genus *Bakuella*, subgenus *B.* (*Bakuella*), and the subgenus *B. (Pseudobakuella)* are rejected by AU tests (*P* = 2e-6, 5e-7, 7e-15, 6e-7, respectively).

### Taxonomic Summary

Subclass Hypotrichia Stein, 1859.

Order Urostylida Jankowski, 1979.

Family Bakuellidae Jankowski, 1979.

Genus *Bakuella* Agamaliev and Alekperov, 1976.

*Bakuella marina* Agamaliev and Alekperov, 1976.

### Improved Diagnosis

The original diagnosis of *B. (Bakuella) marina* Agamaliev and Alekperov, 1976, was only based on silver impregnation specimens. Upon detailed life observations of Chinese population, the important characteristics of cortical granules were supplemented, and the diagnosis was improved as follows: Body in life usually 150–200 × 35–60 μm (present work), sometimes 230–310 μm long, with elongated body shape; 28–51 adoral membranelles; 23–46 left and 32–63 right marginal cirri; 3 frontal, 1–3 parabuccal, 2–5 buccal, and 5–11 frontoterminal cirri; midventral complex composed of 4–12 midventral pairs and 4–8 midventral rows, terminating slightly ahead of transverse cirri; 4–11 transverse cirri formed a short row near the posterior end of cell; 3 bipolar dorsal kineties; caudal cirri lacking; more than 100 macronuclear nodules, 4–10 micronuclei; cortical granules colorless, approximately 1 μm in diameter, formed in irregular short rows, distributed on both ventral and dorsal side; 1 contractile vacuole positioned behind proximal end of adoral zone on the left side.

### Deposition of Voucher Slides

Eight voucher slides (ZR20190826A–H) with protargol-stained specimens were deposited in the Laboratory of Hydrobiology, Hebei University, China. Two slides (ZR20190826I, J) were deposited in the Laboratory of Protozoology, Ocean University of China.

### Gene Sequence

Small subunit ribosomal gene sequence deposited in GenBank under the accession number MT811784.

### Morphological Description

**FIGURE 3:**
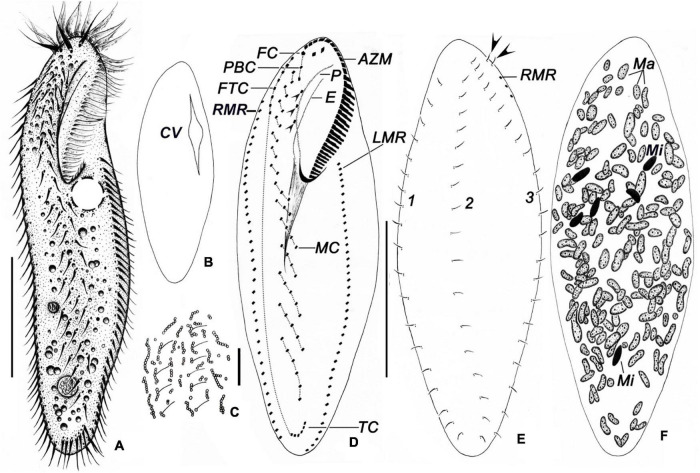
*Bakuella (Bakuella) marina* in life **(A–C)** and after protargol impregnation **(D–F)**. **(A)** Ventral view of a representative individual. **(B)** Showing the collecting canals of the contractile vacuole. **(C)** Arrangement of cortical granules on the dorsal side. **(D–F)** Infraciliature of ventral **(D),** dorsal sides **(E),** nuclear apparatus **(F)**, and arrows in panel **(E)** show the extra basal body pairs. AZM, adoral zone of membranelles; CV, contractile vacuole; E, endoral; FC, frontal cirri; FTC, frontoterminal cirri; LMR, left marginal row; Ma, macronuclear nodules; Mi, micronuclei; MC, midventral complex; P, paroral; PBC, parabuccal cirri; RMR, right marginal row; TC, transverse cirri; 1–3, dorsal kineties 1–3. Scale bars = 50 μm **(A,B,D)**; 10 μm **(C)**.

**FIGURE 4:**
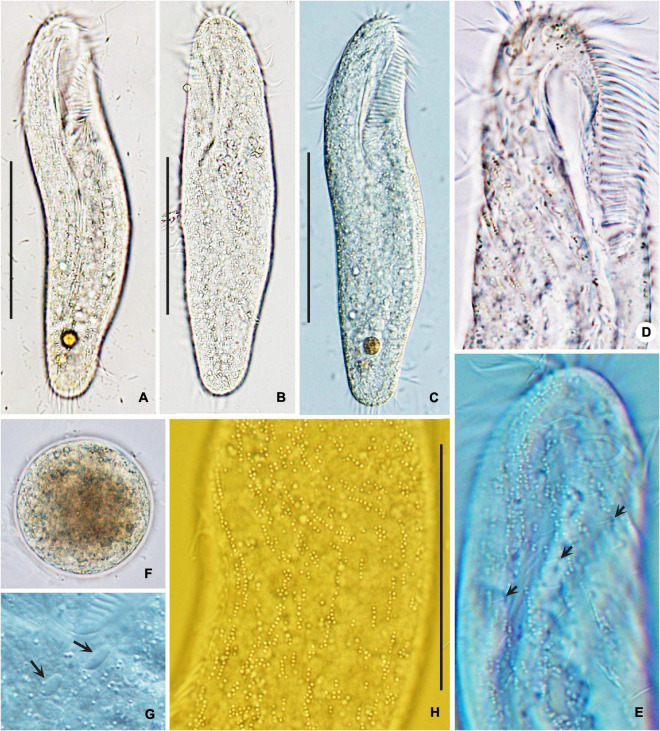
Photomicrographs of *Bakuella (Bakuella) marina* from life in bright filed **(A,B,F)** and differential interference contrast microscopy **(C–E,G,H)**. **(A–C)** Ventral **(A,C)** and dorsal **(B)** views, showing elliptical body shape. **(D,E,H)** Ventral **(D)** and dorsal **(E,H)** views of the anterior body portion, showing the distribution of cortical granules. Dorsal bristles are marked by arrows **(E)**. **(F)** Mature cyst. **(G)** Micronuclei (arrows). Scale bars = 50 μm **(A–C)**; 80 μm **(H)**.

**FIGURE 5:**
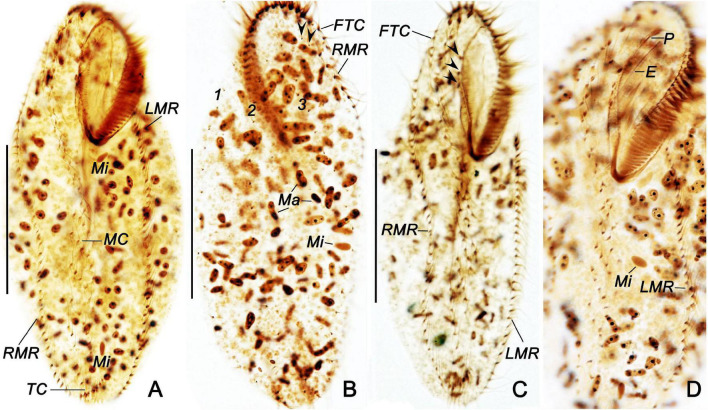
*Bakuella (Bakuella) marina* after protargol staining **(A–D)**. **(A,C,D)** General ciliature on the ventral side; arrowheads indicate the buccal cirri. **(B)** Dorsal side; arrowheads show the basal body pairs. E, endoral; FTC, frontoterminal cirri; LMR, left marginal row; Ma, macronuclear nodules; Mi, micronuclei; MC, midventral complex; P, paroral; RMR, right marginal row; TC, transverse cirri; 1–3, dorsal kineties 1–3. Scale bars = 50 μm.

Body size *in vivo* 150–200 × 35–60 μm, 111–184 × 35–77 μm after protargol staining. Body outline elongates elliptically with both ends rounded, posterior slightly narrowed (Figures 3A, 4A–D). Ratio of length to width is approximately 3.8:1 *in vivo*, as opposed to 2.4:1 in protargol preparations; dorsoventrally flattened at a ratio of approximately 2:1; body flexible and slightly contractile. More than 100 macronuclear nodules are scattered throughout cytoplasm (Figures 3F, 4G, 5B). On average, five large (4–10 × 2–3 μm) micronuclei are also scattered (Figures 3F, 5A,B,D). Contractile vacuole is positioned usually near the posterior end of adoral zone at approximately 40% of body length, on the left side; approximately 25 μm in diameter, contracted at intervals of approximately 6–7 s, collecting canals visible during diastole (Figures 3A,B). Cortical granules colorless, spherical, approximately 1 μm in diameter, formed in irregular short rows along cirral rows and dorsal kineties (Figures 3C, 4A,B,D,E,H). Cytoplasm is colorless to grayish in bright field, with food vacuoles sometimes containing diatoms. Resting cysts are colorless, spherical, in life approximately 45 μm across; the cyst does not show a special surface structure; that is, the wall is smooth (Figure 4F); glides rapidly on microscope slide and soil particles showing great flexibility.

AZM is continuous and extends slightly on left body margin forming a question mark (Figures 3A,D, 4D, 5A–D). Adoral zone occupies on average approximately 40% (30–43%) of body length in stained specimens, approximately 36% *in vivo*, composed of 28–40 (on average 35) membranelles (Table 1). Both endoral and paroral are long, obviously curved, and almost equal in length (paroral and endoral approximately 35.5 and 33.2 μm on average, respectively), intersecting at the level of the posterior-most buccal cirrus (Figure 3D). Consistently there are three enlarged frontal cirri, cilia of which are approximately 17 μm long *in vivo*; 1–3 parabuccal cirri; 2–5 (on average 3) buccal cirri arranged in a longitudinal row close to the paroral; 5–10 (on average 7) frontoterminal cirri below distal end of adoral zone. Midventral complex is approximately 80% down the length of the body and comprised 5–10 midventral pairs arranged in a typical zigzag pattern and 5–8 midventral rows, each of which comprised 3–8 cirri (Figures 3A,D, 5A,C,D). Four to eight transverse cirri formed a short pseudorow near the posterior end of cell with cilia 15–20 μm long. One left and one right marginal row are composed of 31–43 and 32–47 cirri, respectively (Figures 3A,D, 5A,C,D).

**TABLE 1:** Morphometric characterization of the Chinese population of *Bakuella (Bakuella) marina.*

Character^a^	Mini	Max	Mean	Med	*SD*	*SE*	CV	*n*
Body length	111.0	184.0	138.6	140.0	17.7	3.6	12.8	25
Body width	35.0	77.0	57.8	58.0	10.0	2.0	17.3	25
Body length: width, ratio	2.0	3.2	2.4	2.4	0.3	0.1	11.1	25
Adoral zone, length	38.0	63.0	50.0	50.0	6.0	1.2	11.9	25
AZM length: body length, ratio	0.3	0.43	0.4	0.4	0	0	6.9	25
Adoral membranelles, number	28.0	40.0	35.2	35.0	2.9	0.6	8.2	25
Paroral, length	24.0	46.0	35.5	35.0	6.5	1.3	18.2	25
Endoral, length	23.0	46.0	33.2	33.0	5.9	1.2	17.8	25
Buccal cirri, number	2.0	5.0	2.9	3.0	0.6	0.1	21.5	25
Parabuccal cirri, number	1.0	3.0	1.6	2.0	0.6	0.1	35.4	25
Frontal cirri, number	3.0	3.0	3.0	3.0	0	0	0	25
Frontoterminal cirri, number	5.0	10.0	7.0	7.0	1.0	0.2	14.0	25
Midventral pairs, number	5.0	10.0	7.4	7.0	1.2	0.2	15.8	25
Midventral rows, number	5.0	8.0	6.8	7.0	0.9	0.2	13.4	25
Transverse cirri, number	4.0	8.0	6.6	7.0	0.9	0.2	13.6	25
Left marginal cirri, number	31.0	43.0	38.1	38.0	3.4	0.7	8.8	25
Right marginal cirri, number	32.0	47.0	39.4	40.0	3.7	0.8	9.5	25
Micronuclei, number	2.0	8.0	5.0	5.0	1.3	0.3	26.6	25
Length of micronuclei	4.0	10.0	6.2	6.0	1.4	0.3	22.3	25
Width of micronuclei	2.0	3.0	2.4	2.0	0.5	0.1	20.3	25
Micronuclei length: width, ratio	1.3	4.0	2.6	2.5	0.6	0.1	21.5	25
Number of dorsal kineties	3.0	3.0	3.0	3.0	0	0	0	25

*^a^All data are based on protargol-stained specimens. Measurements in μm.*

*CV, coefficient of variation in %; Max, maximum; Mean, arithmetic mean; Med, median value; Min, minimum; n, number of cells measured; SD, standard deviation; SE, standard error of arithmetic mean.*

Three bipolar dorsal kineties with bristles are approximately 5 μm long *in vivo*, composed of approximately 20 dikinetids in each kinety. There are two “extra” dikinetids in front of the right marginal row (Figures 3E, 4E, 5B).

### Morphogenesis During Binary Fission

#### Stomatogenesis

**FIGURE 6:**
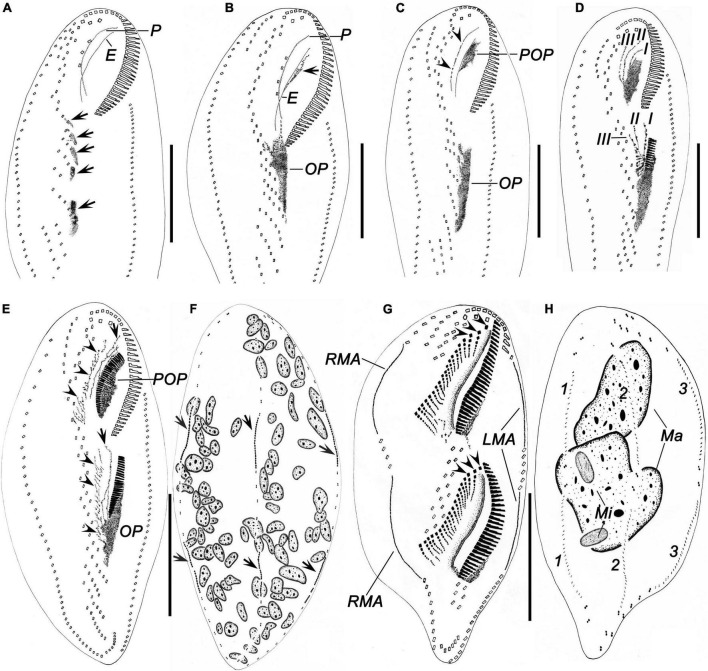
Morphogenesis of *Bakuella (Bakuella) marina* after protargol impregnation. **(A)** Ventral view of a very early divider, showing newly formed group of basal bodies (arrows). **(B,C)** Ventral view of early dividers, showing the *de novo* formation of the proter’s oral primordium (arrow in panel **B**), the cirri in midventral complex that do not join the construction of the opisthe’s oral primordium, and the disintegrating parental paroral (arrowheads in panel **C**). **(D)** Showing the frontoventral–transverse cirral anlagen I, II, and III were formed in an early divider. **(E,F)** Ventral **(E)** and dorsal **(F)** view of a specimen in early stage; arrowheads in panel **(E)** mark the frontoventral–transverse cirral anlagen; arrows mark the anlage I; arrows in panel **(F)** show the dorsal kineties anlagen originating intrakinetally. **(G,H)** Ventral and dorsal views of a middle divider, to demonstrate the frontoventral–transverse cirral anlagen differentiate into cirri; arrowheads in panel **(G)** denote the newly formed frontal cirri. E, endoral; I–III, frontoventral–transverse cirral anlagen I–III; LMA, left marginal row anlagen; Ma, macronuclear nodules; Mi, micronuclei; OP, opisthe’s oral primordium; P, paroral; POP, proter’s oral primordium; RMA, right marginal row anlagen; 1–3, dorsal kineties 1–3. Scale bars = 50 μm.

**FIGURE 7:**
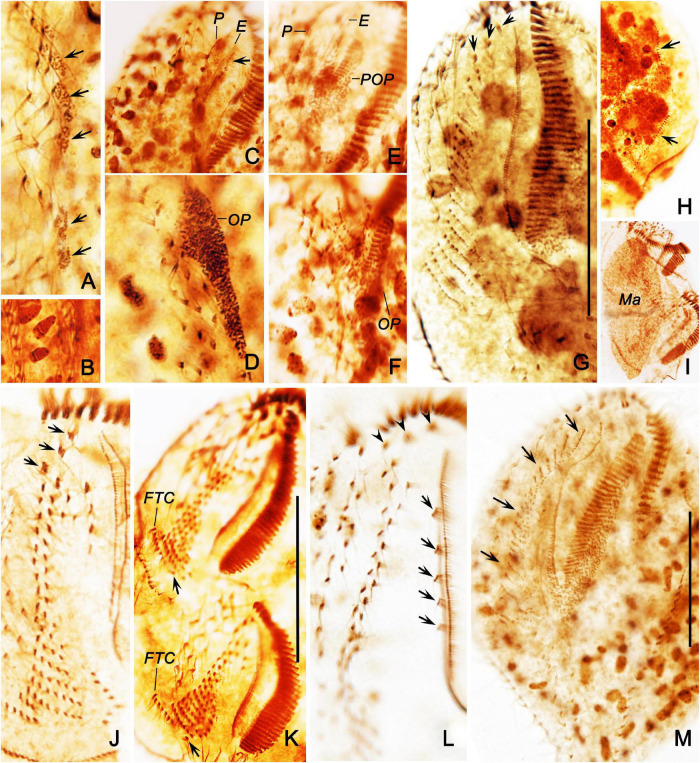
Morphogenesis of *Bakuella (Bakuella) marina* after protargol staining. **(A)** Ventral view of a very early divider to show the newly formed group of basal bodies (arrows). **(B)** Showing the macronuclear nodules with replication band. **(C,D)** Ventral views of the same early divider, to show the *de novo* formation of the proter’s oral primordium (arrow in panel **C**), and the opisthe’s oral primordium **(D)**. **(E,F)** Ventral views of the same early divider, to show the oral primordia of the proter and opisthe differentiate into new membranelles. **(G)** Showing the frontoventral–transverse cirral anlagen differentiate into cirri; arrows mark the newly formed frontal cirri. **(H)** Indicate the dorsal kineties anlagen (arrows) occur intrakinetally. **(I)** Showing the macronuclear nodules fused into a single mass. **(J)** Ventral view of a divider in middle stage, to show the newly formed frontal cirri (arrows). **(K)** Showing the newly formed frontoterminal cirri (FTC) and the rightmost transverse cirri (arrows). **(L)** Showing the new frontal (arrowheads) and buccal (arrows) cirri. **(M)** Ventral view of an early reorganizer; arrows indicate the newly formed frontoventral–transverse cirral anlagen. E, endoral; FTC, frontoterminal cirri; Ma, macronuclear nodules; OP, opisthe’ oral primordium; P, paroral; POP, proter’s oral primordium. Scale bars = 50 μm.

**FIGURE 8:**
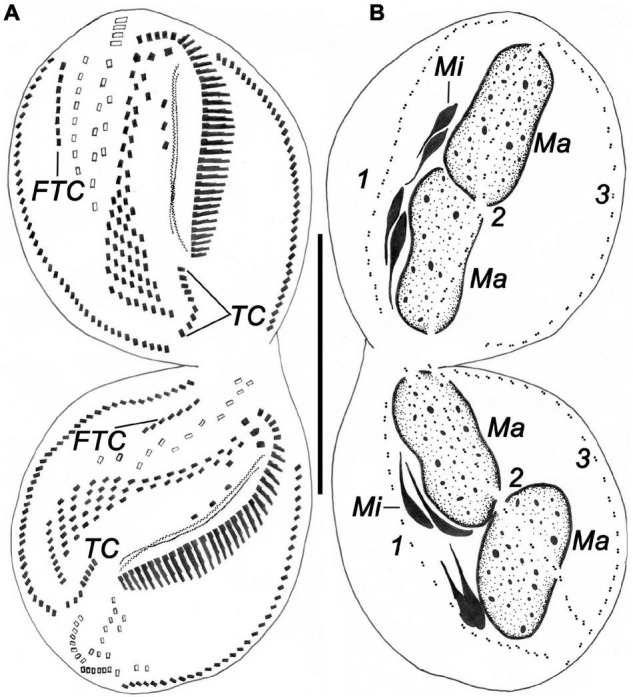
Ventral **(A)** and dorsal **(B)** views of a late divider of *Bakuella (Bakuella) marina* after protargol impregnation showing all ciliary organelles are about in their final positions. FTC, frontoterminal cirri; Ma, macronuclear nodules; Mi, micronuclei; TC, transverse cirri; 1–3, dorsal kineties 1–3. Scale bars = 50 μm.

The oral primordium (OP) in both proter and opisthe develops *de novo*. In the opisthe, stomatogenesis starts with the appearance of several small groups of basal bodies immediately left to the left midventral rows (Figures 6A, 7A); these basal bodies then merge by further proliferation of basal bodies forming a wedge-shaped OP (Figures 6B, 7D,F). In the proter, OP commences with the formation of some basal bodies along the parental endoral and then becomes larger because of further proliferation of basal bodies (Figures 6B,C, 7C,E). Later, several membranelles are formed in the anterior portion in the oral primordia (Figure 7E). Simultaneously, the parental undulating membranes (UMs) are resorbed in the proter, and the UM anlagen (anlage I) of the proter and opisthe are formed to the right of the oral primordia (Figure 6D). Finally, with the new membranelles differentiate in a posteriad direction, the new adoral zone of the membranelles (AZM) is formed in both daughter cells. Note that the parental AZM is entirely replaced by the newly formed one (Figures 6E,G, 7E,J,K, 8A. UMs anlage splits longitudinally into the endoral and paroral in each daughter cell (Figures 7J–L).

#### Development of the Frontal, Midventral, and Transverse Cirri

In the early stage, three frontoventral–transverse cirral anlagen are formed to the right of the OP in both daughter cells (Figure 6D). Anlage I (UM anlage) possibly originates from the OP. Subsequently, all other frontoventral–transverse cirral anlagen appear (Figure 7G); the cirri of the parental midventral complex are not involved (Figure 6E). Then, frontoventral–transverse cirri differentiate during middle and late stages (Figures 6G, 7J). Finally, anlage I contributes the left frontal cirrus; anlage II forms the middle frontal cirrus and the buccal cirri; anlage III produces the right frontal cirrus and the parabuccal cirri; anlage IV and on average the next six anlagen form the midventral pairs (deduced from morphometric data, *n* = 4); anlagen n–1 and on average the six previous anlagen develop the midventral rows (deduced from morphometric data, *n* = 4); anlage n forms the frontoterminal cirri. Usually, approximately seven rightmost anlagen each develop one transverse cirrus, respectively (Figures 6G, 7G,K, 8A). As in many other hypotrichs, reorganization of this species resembles the development of the proter during cell division (Figure 7M).

#### Development of Marginal Rows and Dorsal Kineties

The formation of the marginal rows and dorsal kineties proceeds in the usual way, that is, two primordia being formed within each row/kinety (Figures 6F,H, 7H, 8B).

#### Nuclear Division

The nuclear apparatus divides in the common way, that is, the macronuclear nodules fuse to a single mass during middle stages and separate again (Figures 6H, 7I, 8B). In very early dividers, a replication band was observed in macronuclear nodules (Figure 7B). The micronuclei divide in a conventional manner (Figures 6H, 8B).

#### Occurrence and Ecology

As mentioned previously, Chinese population of *B. (Bakuella) marina* was found in hypersaline waters (salinity approximately 50‰). The population grows well under laboratory conditions (salinity 50‰, temperature 25°C). *B. (Bakuella) marina* feeds on diatom and bacteria. When conditions are not conducive, the species is able to quickly form a cyst (Figure 4F). By using nonflooded Petri dish method (Foissner et al., 2002), *B. (Bakuella) marina* was also isolated from the soil samples collected near the water sampling site, which shows that this species can also survive in high-saline soils. The salt tolerance experiment shows that the species could adapt to the salinity of 10–60‰.

## Discussion

### Comparison of Chinese Population With Previous *Bakuella (Bakuella) marina* Populations

**FIGURE 9:**
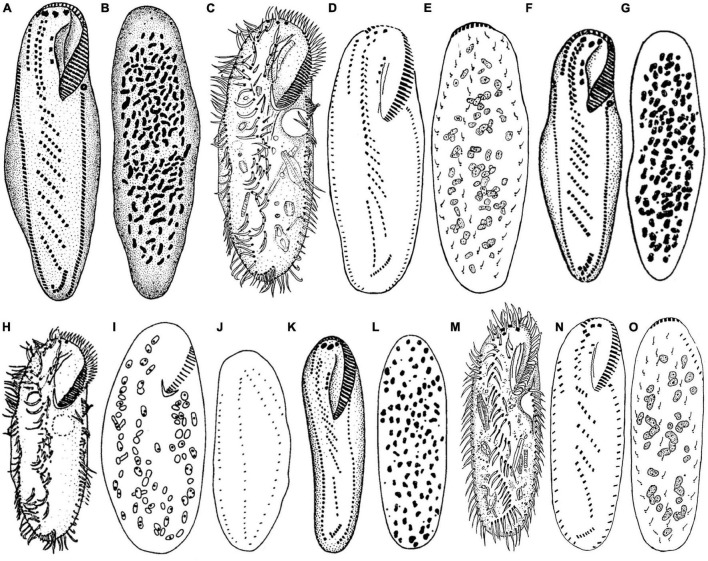
Comparative illustrations of some populations. **(A,B)** From Agamaliev and Alekperov (1976). **(C,H**–**J)** From Wilbert (1986). **(D,E,M**–**O)** From Song et al. (1992). **(F,G,K,L)** From Alekperov (1982). Panels **(A,B)** = 130 μm. Panels **(C–E)** = 230–310 μm. Panels **(F,G)** = size not indicated. Panels **(H–J)** = 210 μm. Panels **(K,L)** = 110–130 μm. Panels **(M–O)** = 91–108 μm.

Berger (2006) provided a detailed review of the taxonomical history of *B. (Bakuella) marina.* This species was first reported by Agamaliev and Alekperov (1976). Later, Alekperov (1982) reported a species *B. (Bakuella) imbricata* isolated from a freshwater reservoir (Djeiranbatansky in Azerbaijan). Thereafter, two further populations of *B. (Bakuella) marina* were recorded by Wilbert (1986) and Song et al. (1992). Based on a detailed comparison of the four known populations (Figure 9 and Table 2), Song et al. (1992) synonymized *B. (Bakuella) marina* and *B. (Bakuella) imbricata* because there was no significant difference in the infraciliature between them. Unfortunately, the above four populations were reported with no detailed live observation information (Berger, 2006).

**TABLE 2:** Comparisons of characteristics of *Bakuella (Bakuella) marina* in present and previous populations.

Character^a^	Present record	Pop 1	Pop 2	Pop 3	Pop 4
Habitat	Hypersaline water and soil	Marine	Saline lake	Freshwater	Saline lake
Body length after protargol impregnation	138.6 (111.0–184.0)	120.0–140.0	266.7 (230.0–310.0)	110.0–130.0	101.4 (91.0–108.0)
Adoral membranelles, number	28.0–40.0	32.0–36.0	34.0–51.0	38.0–40.0	28.0–36.0
Buccal cirri, number	2.0–5.0	3.0–4.0	2.0–5.0	4.0–5.0	2.0–4.0
Parabuccal cirri, number*	1–3	Likely 1	Likely 2	Likely 1	Likely 1
Frontoterminal cirri, number	5.0–10.0	About 9.0	5.0–11.0	About 9.0	5.0–9.0
Midventral pairs, number	5.0–10.0	About 12.0	4.0–12.0	4.0–8.0	4.0–5.0
Midventral rows, number	5.0–8.0	About 10.0	5.0–8.0	5.0–7.0	4.0–5.0
Transverse cirri, number.	4.0–8.0	About 10.0	5.0–11.0	About 7.0	5.0–9.0
Left marginal cirri, number	31.0–43.0	52.0–56.0	23.0–53.0	32.0–35.0	25.0–33.0
Right marginal cirri, number	32.0–47.0	55.0–60.0	34.0–63.0	40.0–42.0	34.0–54.0

*^a^ Measurements in μm.*

*Pop 1, from Agamaliev and Alekperov (1976), wet silver impregnation; Pop 2, population of Wilbert (1986), data from Song et al. (1992), protargol impregnation; Pop 3, B. imbricata population from Alekperov (1982), wet silver impregnation; Pop 4, from Song et al. (1992), protargol impregnation.*

*Number of parabuccal cirri in Pop 1–4 inferred according to the drawings, respectively.

The present Chinese population was defined as *B. (Bakuella) marina* on the basis of the following features: body size *in vivo* 150–200 × 35–60 μm, with 2–5 buccal and 1–3 parabuccal cirri, 5–10 frontoterminal cirri, 4–8 transverse cirri, midventral complex comprised 5–10 cirral pairs and 5–8 rows, 31–43 and 32–47 cirri in left and right marginal rows, respectively, and 28–40 adoral membranelles. The above features correspond very well with those of previous populations (Table 2). Because of the lack of detailed living data, whether cortical granules are present in *B. (Bakuella) marina* remains unknown. The present work shows that it has colorless cortical granules.

### Morphogenetic Pattern of *Bakuella (Bakuella) marina*

Previous studies indicate that *Bakuella* is ontogenetically diverse (Chen et al., 2020b). Based on the partial or complete reorganization of the proter’s AZM during cell division, there are two morphogenetic types in this genus (Song and Shao, 2017). In *B. (Bakuella) pampinaria*, *B. (Bakuella) subtropica*, *B. (Pseudobakuella) guangdongica*, and *B. (Pseudobakuella) salinarum*, the parental AZMs are renewed completely (Berger, 2006; Chen et al., 2013; Li et al., 2021); however, in *B. (Bakuella) edaphoni*, *B. (Bakuella) granulifera*, and *B. (Bakuella) pampinaria*, the parental AZM is reorganized only in the proximal part (Song et al., 1992; Berger, 2006; Chen et al., 2020b). Based on the present observation, the fate of old AZM in *B.* (*Bakuella*) *marina* is similar to the former.

It is worth mentioning that there are also some other tiny differences in the destiny of the dedifferentiated parental UMs among different species within *Bakuella*: in *B. (Bakuella) marina* and *B. (Bakuella) subtropica*; the disintegrated old paroral membrane is likely not involved in the formation of the OP. In another known species, *B. (Bakuella) edaphoni*, the new UM anlage originates from the disintegration and reorganization of the parental UMs. In *B. (Bakuella) pampinaria*, only the paroral membrane is disintegrated to form the UM anlage, whereas the proliferated basal bodies at the anterior end of the endoral membrane likely participate in the formation of the proter’s OP (Mihailowitsch and Wilbert, 1990; Eigner and Foissner, 1992; Song et al., 1992).

The origin of the frontoterminal cirri is fully understood: in *Bakuella (Bakuella) marina*, as well as *B. (Bakuella) granulifera, B. (Bakuella) pampinaria, B. (Bakuella) subtropica, B. (Pseudobakuella) guangdongica*, and *B. (Pseudobakuella) salinarum*, the frontoterminal cirri are produced from the anterior end of the rightmost frontoventral-transverse (FVT) anlage, whereas in *B. (Bakuella) edaphoni*, its frontoterminal cirri seem to be formed from the penultimate FVT anlage (Eigner and Foissner, 1992; Song et al., 1992; Berger, 2006; Chen et al., 2013, 2020b; Li et al., 2021).

### Phylogenetic Analyses

The SSU rDNA sequence (MT811784) of the type species of genus *Bakuella* was provided for the first time in this article. Our phylogenetic tree indicates that these six *Bakuella* species fall into three clades, and the AU test rejects the monophyly of the genus *Bakuella*. Similarly, the result of AU test also rejects the monophyly of the family Bakuellidae, subgenus *B.* (*Bakuella*), and the subgenus *B.* (*Pseudobakuella*), respectively. The above results are consistent with those of the previous studies (Chen et al., 2013; Fan et al., 2014; Jo et al., 2015; Lv et al., 2015; Lyu et al., 2018; Ma et al., 2021).

As mentioned previously, there are currently two morphogenetic patterns in genus *Bakuella* based on the fate of the parental AZM. It should be pointed out that the species that belong to the same morphogenetic pattern are not clustered together in the tree. *B. (Bakuella) marina*, *B. (Bakuella) subtropica*, and *B. (Pseudobakuella) guangdongica* clustered in two clades; *B. (Bakuella) granulifera* and *B. (Pseudobakuella) guangdongica*, however, clustered in one clade (Figure 2). The above morphogenetic and molecular information suggests that *Bakuella* should be subdivided. However, because of the fact that only four species (*B. (Bakuella) marina*, *B. (Bakuella) granulifera*, *B. (Pseudobakuella) guangdongica*, and *B. (Bakuella) subtropica*) in *Bakuella* are available on both their morphogenetic and molecular data, it is still premature to divide *Bakuella* currently. Further investigations on both the morphogenetic and molecular information of more congeners in *Bakuella* and related taxa (especially *Anteholosticha*) are needed in order to establish a robust classification of genus *Bakuella* and family Bakuellidae.

## Conclusion

The present study once again shows the nonmonophyly of family Bakuellidae, genus *Bakuella*, and subgenus *B. (Bakuella)*. Because some morphological features (such as bicorona or midventral rows in the urostyloidea) have evolved convergently (Berger, 2006), it is not enough to study the phylogeny of hypotrichs based solely on morphological characteristics, and it is necessary to integrate molecular, ontogenetic, and even ultrastructural information to establish a reasonable phylogenetic relationship (Dong et al., 2020). Therefore, there is an urgent need for molecular and morphogenetic information of *Bakuella* and its related species to establish a reliable taxonomic system of genus *Bakuella* and family Bakuellidae.

## Data Availability Statement

The datasets presented in this study can be found in online repositories. The names of the repository/repositories and accession number(s) can be found below: https://www.ncbi.nlm.nih.gov/, MT811784.

## Author Contributions

FL sampled the material and planned the study. RZ, QZ, and LT conducted indoor culture of ciliate population and silver staining, and generated the molecular data. RZ conducted the phylogenetic and network analysis, and wrote the initial version of the manuscript. JL, FL, and YZ revised the manuscript. All authors contributed to the discussion of the results and preparation of the final manuscript, and read and approved the final manuscript.

## Conflict of Interest

The authors declare that the research was conducted in the absence of any commercial or financial relationships that could be construed as a potential conflict of interest. The handling Editor declared a past collaboration with the authors.

## Publisher’s Note

All claims expressed in this article are solely those of the authors and do not necessarily represent those of their affiliated organizations, or those of the publisher, the editors and the reviewers. Any product that may be evaluated in this article, or claim that may be made by its manufacturer, is not guaranteed or endorsed by the publisher.
